# Perspectives on existential loneliness. Narrations by older people in different care contexts

**DOI:** 10.1080/17482631.2023.2184032

**Published:** 2023-03-05

**Authors:** Helena Larsson, Ingela Beck, Kerstin Blomqvist

**Affiliations:** a Faculty of Health Sciences, Kristianstad University, Kristianstad, Sweden; b The Research Platform for Collaboration for Health, Kristianstad University, Kristianstad, Sweden; c The Institute for Palliative Care, Region Skane and Lund University, Lund, Sweden; d Department of Clinical Sciences Lund, Division of Oncology, Faculty of Medicine, Lund University, Lund, Sweden

**Keywords:** Existential loneliness, older people, care contexts, interviews, theory of suffering

## Abstract

The aim was to explore existential loneliness in different long-term care contexts as narrated by older people. A qualitative secondary analysis was performed of 22 interviews with older people in residential care, home care, and specialized palliative care. The analysis started with naive reading of interviews from each care context. As these readings showed similarity with Eriksson’s theory of the suffering human being, the three different concepts of suffering were used as an analytic grid. Our result indicates that suffering and existential loneliness are interrelated for frail older people. Some situations and circumstances that trigger existential loneliness are the same in the three care contexts while others differ. In residential and home care, unnecessary waiting, not feeling at home and not being encountered with respect and dignity can trigger existential loneliness while seeing and hearing others suffering can give rise to existential loneliness in residential care. In specialized palliative care, feelings of guilt and remorse are prominent in relation to existential loneliness. In conclusion, different healthcare contexts have various conditions for providing care that meet the existential needs of older people. Hopefully our results will be used as a basis for discussions in multi-professional teams and among managers.

## Introduction

Old age is a time in life that often evokes existential thoughts (Sjöberg et al., 2018). As many older people become frail and in need of care, it is likely that health care professionals will become increasingly important for the older person in moments when existential issues come to mind (Norell Pejner et al., 2015). However, studies show that health care professionals experience that it is challenging to face existential concerns and satisfy existential needs (Sundström et al., 2018; Udo, 2014) and that the care context is important for how existential needs are addressed (Sundström et al., 2019). One existential issue is existential loneliness. Existential loneliness is described as a deeper feeling of loneliness (Ettema et al., 2010), as intertwined with other forms of loneliness (Applebaum, 1978), and as a human condition (Yalom, 1980). Recently, empirical studies have been conducted regarding existential loneliness among older people (Carr & Fang, 2021; Sjöberg et al., 2018) and significant others (Larsson et al., 2017). In addition, health care professionals have provided their views about older persons’ existential loneliness (Sundström et al., 2018). Studies show that there are similarities in the origins of existential loneliness, but also that there are differences. In order to gain a deeper understanding of existential loneliness, it is important to explore not only existential loneliness as a phenomenon but also in relation to specific care contexts where older persons are most often cared for.

A recently published compilation of 144 qualitative studies on loneliness describes three forms: social loneliness, emotional loneliness, and existential loneliness (Mansfield et al., 2019). Social loneliness is described as a longing for companionship with other people (Perlman & Peplau, 1998; Weiss, 1987). Emotional loneliness is described as the feeling of loneliness even though there are people around or when it is not possible to share one’s thoughts with another person (Weiss, 1987). Existential loneliness is summarized as a form of loneliness that emerges especially when people are confronted with life-threatening illness, trauma, dying, and death (Mansfield et al., 2019). Although different forms of loneliness are intertwined and difficult to distinguish in human life, an attempt has been made in a recent qualitative study where narrations about loneliness among older people in long-term care facilities were analysed in relation to the three different forms of loneliness. The existential dimension of loneliness was interpreted as living in nothingness and as part of lonely humanity, i.e., a lonely life and a lonely death (Jansson et al., 2022). Existential loneliness is described by the existential psychologist Irvin Yalom (1980) as a deep form of loneliness, as a condition of life and as part of being human. He explains that life involves events when all humans face existential concerns. These events are referred to as the ultimate concerns and are about the inevitability of death, our need for freedom, our need to feel community and togetherness, and the search for meaning. Confronting and facing such events in life is inevitable (Yalom, 1980) and according to Applebaum (1978), existential loneliness arises in transitional phases in life. Existential loneliness is referred to as an experience and as a process that can lead to growing as a person, intellectually and spiritually (Ettema et al., 2010; Moustakas, 1961). However, existential loneliness seems to emerge when humans realize that they are alone in the world, even though there are people around (Moustakas, 1961; Yalom, 1980). Existential loneliness has been studied in a literature review (Ettema et al., 2010) and is described as a fundamental feeling of loneliness, intertwined with other forms of loneliness and with other experiences such as anxiety and meaninglessness. A concept analysis (Bolmsjö et al., 2018) describes existential loneliness as an immediate awareness of abandonment that appears especially in moments when one realizes one’s own mortality or in crises. As a result of this awareness, emotions such as sadness, hopelessness, anxiety, and meaninglessness are experienced.

A few empirical studies have been conducted on health care and show that existential loneliness arises when a person is in a vulnerable situation. Examples of vulnerability are when a person suffers from aphasia after a stroke (Nyström, 2006), lives with HIV (Mayers & Svartberg, 2001), moves to retirement living (Carr & Fang, 2021), or is at the end of their life (Sand & Strang, 2006). In addition, a qualitative study on the meaning of existential loneliness among frail older people has been conducted (Sjöberg et al., 2018). The study included frail older people in need of long-term care received at home, in a residential care facility or by specialized palliative care. The study shows that existential loneliness was associated with feeling trapped in a frail body, being met with indifference, having no one to share life with and lacking purpose and meaning in life (Sjöberg et al., 2018). In order to better encounter and ease the existential loneliness of older people, it is important to build/develop knowledge about situations and circumstances in different care contexts that can lead to existential loneliness. From the above, it appears that there is no generally accepted definition of existential loneliness. However, what most philosophers and researchers seem to agree on is that existential loneliness is a deep form of loneliness, a feeling that can come and go and be more or less intense and arises in vulnerable situations such as transitions and crises in life.

Older people are provided with care from many different professionals, and globally, care is structured in several different ways (WHO, 2021) and can take place in a number of different places. For some people, the most important matter is to stay in their own home environment for as long as possible, which leads to care providers having to encounter persons with complex care needs. The needs are not only physical but also of an existential nature and relate to the fact that life is coming to an end (Sjöberg et al., 2018; van Wijngaarden et al., 2015). Another form of care is provided in residential care facilities. A meta-synthesis (Vaismoradi et al., 2016) shows how older people wished for a homelike and secure place where they would receive good care. In Sweden, the focus for residential care changed in 1992 and the intention was that a residential care facility should be more like the person’s own home rather than an institution (Sveriges riksdag 1990/91:14). Palliative care is yet another form of care for people whose existence is threatened by progressive, incurable illness or injury. A central goal of palliative care is to achieve, support, preserve, and enhance the best possible quality of life (Radbruch & Payne, 2010). Palliative care is provided in different ways in different countries and older people are not always offered palliative care when they need it (Radbruch & Payne, 2010). In Sweden, home care and specialized palliative care can be provided in a person’s own home and when care can no longer be provided in a home environment or if there is a lack of security, residential care, or specialized palliative in-patient care may be considered. A study where health care professionals in home, residential, hospital, and palliative care settings were interviewed concludes that the context of care matters and influences how professionals view existential aspects such as existential loneliness. The study points to how different contexts’ intentions and foundations of value influence the professionals’ opportunities and abilities to focus on patients’ existential concerns (Sundström et al., 2018).

Research indicates that older people’s existential loneliness is not being adequately met (Sjöberg et al., 2018; Österlind et al., 2017). This implies that health care professionals have an important role in encounters with the older persons to whom they provide care. However, research reveals that health care professionals experience that it is challenging to face existential loneliness (Sundström et al., 2018) and that the care context is important for how existential loneliness is addressed (Sundström et al., 2019). One way to gain a closer understanding of how existential loneliness is experienced is to look specifically at the different care contexts where older people are most often cared for. This knowledge can provide insight into how targeted interventions aiming to alleviate existential loneliness in different care contexts can be planned and conducted. Therefore, the aim was to explore existential loneliness in different care contexts as narrated by older people.

## Materials and methods

### Design

The study started with an explorative phase and during the process of analysis Katie Eriksson’s theory of suffering (Eriksson, 1994/2006) was used as a theoretical framework. The study is part of a larger research project, the LONE study, RR2–10.2196/1307 (Edberg & Bolmsjö, 2019). The overall purpose of the research project was to explore existential loneliness from different perspectives: older persons, their relatives, health care professionals, family care advisors, volunteers, and health care managers. The present study has its starting point in one of the previous studies conducted in the LONE study with frail older persons’ narrations about experiences of existential loneliness (Sjöberg et al., 2018). That study focused on the meaning of existential loneliness regardless of care context; in this study; however, we aimed to explore experiences of existential loneliness in different care contexts by performing a secondary analysis of the interviews. The included care contexts for this study come from different municipalities in the southern part of Sweden and cover residential care, home care, and specialized palliative care.

### Context and setting

Approximately one million Sweden’s population are over 75 years old (SCB, Central Bureau of Statistics, 2018). The formal care of older people in Sweden is largely provided by employees in 290 municipalities. Older people can apply for various kinds of support in their home and when their needs can no longer be provided for at home, residential care can be offered. A home help officer conducts a needs assessment to decide the appropriate type of support for each person. In home care, care can be provided by home care workers, nurse assistants, registered nurses, primary health care, and at a hospital for certain treatments. Specialized palliative care requires a remittance from a physician and the care can be provided in the person’s own home or at an in-patient ward. In home care and specialized palliative care, the care is provided at predetermined hours usually combined with the possibility of pressing an alarm or calling if help is needed between these times. In residential care, the professionals are close by and can provide care whenever the help is needed. In all three care context care is provided round the clock. Most professionals in home care and residential care are home care workers, nurse assistants, and registered nurses who can offer health care, including medication. In specialized palliative care, the professionals are mainly registered nurses and the care is provided by a whole team led by a physician.

### Data collection and procedure

Individual in-depth interviews with frail older persons (*n* = 22) served as a basis for this secondary analysis. In the original study (Sjöberg et al., 2018), 23 older persons were interviewed. As one of them did not talk about existential loneliness, this interview was not a part of the data in the present study. The older persons were recruited by a designated contact person at each care unit who provided oral and written information about the study. Inclusion criteria were being 75 years or older, frail, and capable of and interested in participating in an interview and talking about experiences of existential loneliness, i.e., a deep form of loneliness. Exclusion criteria were having cognitive decline and not speaking the Swedish language. The concept “frail older persons” was defined as being 75 years or older and dependent on long-term care or services related to health problems. If the older person gave their permission, their name was communicated to one of the co-workers who contacted the older person, asking them whether they wanted to participate in the study. Among those who showed interest in participating, there were three who later declined because of deteriorating health or because they had changed their minds. Among the 22 included older persons, 12 were men and 10 were women, aged 76–101 years (median = 85 years). They received care in three different contexts: residential care (*n* = 7), home care (*n* = 8), and specialized palliative care (*n* = 7). For a description of the sample, see Table 1. For a more detailed description of the participants, see Sjöberg et al. (2018).

**Table I.** Description of the sample.

**In total**	Age range 76–101 years, median 85 years
Participants	n = 22
Women	n = 10
Men	n = 12
**Residential care**	Age range 80–101 years, median 94 years
Women	n = 5
Men	n = 2
**Home care**	Age range 78–95 years, median 84.5 years
Women	n = 3
Men	n = 5
**Specialized palliative care**	Age range 76–89 years, median 78 years
Women	n = 2
Men	n = 5

The interviews were conducted by two researchers, of whom one is one of the authors of this study, IB (see Sjöberg et al., 2018). The time and place for the interview were decided by the older person. The interviews lasted between 36 and 147 minutes (median = 61 min) and were conducted between February 2015 and July 2016. The interviews were of narrative nature, with open-ended questions to stimulate reflection. The interviews started with a conversation to familiarize with each other. After that, a conversation about their experiences of loneliness in general was introduced as follows: There are many ways to experience loneliness. You can feel lonely together with others, and you can want to be alone and even long to be alone. How do you think about loneliness? The interview then continued by introducing existential loneliness with the following question:

We are particularly interested in your experiences of a deeper feeling of loneliness, called existential loneliness, a feeling that can come and go and be more or less intense. Could you please describe a situation when you have experienced existential loneliness?

Additional follow-up questions such as “Can you tell us more about this?” were used to deepen the participants’ narrations. The interviews were digitally recorded and transcribed verbatim.

### Ethical considerations

The study was approved by the Ethical Review Board, Lund, Sweden (Reg. no. 2014/652). The older persons gave both oral and written consent to participate in the study. It was made clear that they could withdraw from the study at any time. The risk was that the frail older persons would be exposed to too much stress during the interviews, and therefore it was important to be sensitive and not force them to say more than they felt was possible. Permission to record the interview was obtained from the informants.

### Analysis

The analysis was conducted in accordance with Merriam and Tisdell (2016) and their description of the “step-by-step process of analysis”. First, the interviews were sorted based on the three care contexts—residential care, home care, and specialized palliative care—and we all, separately, read the interviews, one care context at a time. The division into three contexts was based on where the participants mainly lived their daily lives and received long-term care—in a residential care facility, in the person’s home with approved assistance from the care community, or in the person’s home with approved specialized palliative care. Within each context, all texts that we agreed concerned existential loneliness were extracted. The extracted narrations covered, for example, limit and vulnerable situations, while narrations about how existential loneliness was eased, or when the interviewees wanted to be alone, were not included. After having read and discussed the material in the group several times, we all agreed that existential loneliness in different care contexts among older persons was linked to different circumstances and situations. For some, existential loneliness was connected to illness, while for others, existential loneliness was linked to the care provided and/or to life itself. During the readings, recurrent in the narrations about existential loneliness was the notion of suffering and this led us further into the theoretical framework by Katie Eriksson (1994/2006) about the suffering human being. According to Eriksson, *suffering of illness* involves experiences in relation to bodily concerns and/or shame and guilt caused by illness and treatment. *Suffering of care* involves experiences in the caring situation such as uncertainty and waiting, condemnation, and reduced dignity. *Suffering of life* involves suffering related to what it means to live, and to be a human being among other human beings. We decided to use her three concepts in the next reading of the narrations—suffering of illness, suffering of care, and suffering of life. The choice to use Eriksson’s theory was thus initially based on the readings of the interviews. We realized that we needed a structure to organize the extensive interview material not only according to three different care contexts but also according to a structure relevant to the descriptions of existential loneliness. These readings revealed similarities between suffering (Bergbom et al., 2021; Eriksson, 1994/2006) and existential loneliness. In addition, we had found in a previous empirical study that existential loneliness could be triggered in encounters with health care (Sjöberg et al., 2018), what Eriksson refers to as suffering of care. The next step was therefore to construct an analytic grid, described by Merriam and Tisdell (2016) as analytical coding, for clustering the extracts about existential loneliness from the three care contexts into Eriksson’s types of suffering; see Table 2. The first author made a first draft where texts in the grid were put together into categories and were given preliminary codes. We thereafter all met again several times to discuss the categorization and the preliminary codes. To be able to distinguish patterns in the material, the categories were compared between the three care contexts. These discussions resulted in several changes, and discussions continued until consensus was reached.

**Table II.** The analytic grid.

	Care contexts:			
Existential loneliness related to:		Residential care	Home care	Specialized palliative care
Suffering of illness				
Suffering of care				
Suffering of life				

## Results

Existential loneliness is presented in relation to the three concepts in Eriksson’s theory (1994/2006) suffering of illness, suffering of care, and suffering of life. Each main paragraph ends with a summary that covers a brief description of our findings about existential loneliness in relation to Eriksson’s theory and between the three care contexts. For an illustration of the findings, see Table 3.

**Table III.** Illustration of the findings.

Care contexts: Existential loneliness related to:	Residential care	Home care	Specialized palliative care
Suffering of illness	Dependent and limited in freedom Shame for who they have become	Dependent and limited in freedom Shame for who they have become	Dependent and limited in freedom Shame for who they have become
Suffering of care	Neglected while waiting for care Undignified and treated like a task to be performed Disrespected and not encountered as an adult Exposed and forced to seeing and hearing others suffering	Neglected while waiting for care Undignified and treated like a task to be performed Disrespected and not encountered as an adult -	- - - -
Suffering of life	Abandoned and forgotten in the loss of close relationships Remorseful over what cannot be undone in life Lack of meaning and purpose in life Not feeling at home-	Abandoned and forgotten in the loss of close relationships Remorseful over what cannot be undone in life Lack of meaning and purpose in life Not feeling at home -	Abandoned and forgotten in the loss of close relationships Remorseful over what cannot be undone in life Lack of meaning and purpose in life - Guilt for how they have lived their lives

### Existential loneliness in relation to suffering of illness

Existential loneliness in relation to suffering of illness contains the participants’ narrations about dependency on others and about shame for who they had become due to illness and treatment. Such narrations were found in all three care contexts.

#### Dependent and limited in freedom

Narrations about dependence and limited freedom were present in all three care contexts. Existential loneliness was found in situations where the participants described how they due to bodily impairments had to *ask* for help, when they felt like a burden to others, when they had to depend on others and their freedom was limited. Narrations from participants receiving *residential care* involved situations where they had to “ring a bell” for the professionals to come. In situations where they knew that the professionals were busy, they expressed ambivalence about whether they should ring on the bell or not. The participants yearned to be able to manage their needs by themselves and be less dependent on others. Likewise, in narrations from participants receiving *home care* the dependency was described in terms of lacking the ability to do what they used to in their own home. The narrations covered situations where someone else performed tasks in their home in a different way than they wished and how this triggered existential loneliness. In narrations from participants receiving *specialized palliative care* the dependency was found in narrations about having to “beg for help” while they wished they could have performed the tasks independently. In the following quote, existential loneliness due to dependency and limited freedom is expressed by a woman receiving residential care:

… I think it would have been better if I could walk and use my legs, now I have to bother them with everything I want to do…they get annoyed…so I lie there and I can’t move…//… Just that I lie here and can’t come down, I can’t walk…//…I’m very afraid of everything…every time I go to bed I have to roll on the bed when they are supposed to help me and I’m afraid of that…//… and then I’ll take them by the wrist and my whole body trembles…and I know that when I go to bed, that’s what’s waiting for me, well…//…when I ask them for a pillow, they just look and say; ‘you’ve got the pillow’ and then they leave again…but I say it doesn’t help, I have to lie here and ask for everything.

(10, woman, residential care)

#### Shame for who they have become

Existential loneliness was found in narrations connected to shame for who they had become due to illness and treatment and was found in all three care contexts. In the participants’ descriptions, they used words such as “horrified” regarding their own incapability. The feeling of shame was expressed by saying “have to sneak out and cry”, meaning that crying is shameful and something they wanted to hide from others:

…it’s this anxiety and horror when you…I mean if you’re seventy-six years old you shouldn’t have to sneak out to the toilet and start crying, because you can’t do anything…//…just that thing of not being able to do anything, right. I mean, if I’m one hundred and eighty metres from the toilet and there are three persons who help me, then I’m ashamed, I don’t want to ask for help, and that’s why I’m ashamed, I’ve always done everything myself, now I can’t do anything.

(16, man, specialized palliative care)

#### In summary

Bodily impairments limit freedom and make people feel like a burden to others. Apart from dependency, shame for who one has become can trigger existential loneliness. The narrations about existential loneliness show similarity with Eriksson’s theory about suffering of illness. The theory relates bodily pain to physical suffering and the shame and humiliation to mental suffering. Existential loneliness was found in all three care contexts in relation to suffering of illness. To constantly have to beg for help restricts people from freely doing *what* they want *when* they want to. The shame is linked to situations where people try to hide who they have become because of their illness.

### Existential loneliness related to suffering of care

Existential loneliness in relation to suffering of care contains the participants’ narrations about having to wait for care, feeling like a task to be performed, not feeling encountered like an adult, and being exposed to others’ suffering. Such narrations were found in residential care and home care but not in specialized palliative care.

#### Neglected while waiting for care

Waiting for care was expressed by participants receiving residential care and home care. Participants receiving *residential care* expressed waiting for care as endless and this made them feel lonely and neglected when not being acknowledged in their needs. This situation revealed a sense of emptiness and uncertainty. The waiting was experienced as being placed in a queue waiting for “their turn”. This was expressed by a man in residential care as follows:

…that’s when I can…feel…alone, when I’m waiting for help…waiting for my turn…there it can be…empty sometimes…//…sometimes it happens that I have really long waiting times.

(7, man, residential care)

Likewise, participants receiving *home care* expressed how they spent their everyday life waiting for and being prepared when the professionals arrived. The waiting led to anger and feelings of not being prioritized and brought experiences of feeling neglected and worthless. To a direct question about a deeper feeling of loneliness, a woman expressed her feelings of endless waiting as “waiting and waiting”:

A: Could you please think back for a moment and recall a time when you have felt deeply alone, had a deep sense of loneliness?

B: …well, I’ve done that sometimes when I’m waiting for them to come and things like that, it almost makes me angry, angry at waiting too long… waiting and waiting and waiting.

(2, woman, home care)

#### Undignified and treated like a task to be performed

This was expressed by participants receiving residential care and home care. Narrations from participants receiving *residential care* and *home care* included experiences of being treated like a task to be performed the way the professionals wanted. Such experiences gave rise to diminished dignity and existential loneliness. Feeling reduced to a task, an object, a part of the machinery, as well as condemnation from professionals, was part of suffering of care. The following quote from a woman in home care who asked for help with a task that was important for her illustrates such experiences:

…then I asked if I could get some help if I washed the laundry myself, to hang, because I have a rack that you can use. No, I couldn’t get that help. Then I know I felt sad…//…Couldn’t they help me hang the laundry if I arrange it myself so that I’ve washed it and it’s just lying there?…I can pick it up too, but I have trouble with the clamps…//… [the professionals] ‘We’re not allowed to do that, we’re not allowed to hang up the laundry’… they follow the procedures…//…then I feel sad…what situation am I in, what should I do, am I really that vulnerable?…// … I’m part of the machinery

(1, woman, home care)

#### Disrespected and not encountered as an adult

This was only expressed by participants receiving residential care and home care. The participants described situations where they felt diminished, not respected and encountered as equal adults and related this to existential loneliness and suffering of care. Participants receiving *residential care* and *home care* referred to events in everyday life such as how they were spoken to or whether their opinion was requested or not. A woman who was cared for in a residential facility expressed that she did not feel met with respect for her wishes nor as an adult when she was not allowed to participate in decisions. She gave as an example when birthdays were celebrated with balloons:

…and then it feels so weird… they arrange parties and stuff, balloons and stuff, I think it’s for kids, not for grown-ups…

(10, woman, residential care)

#### Exposed and forced to seeing and hearing others suffering

This was only expressed by participants receiving residential care. Living together with others as in the case for those living in *residential care* made seeing and hearing other persons’ suffering unavoidable. A man who received residential care expressed how he felt sad and distressed when seeing and hearing other people around who were suffering and how this in turn gave rise to experiences of existential loneliness:

…to see old people sitting and crying…*it feels so hard*…//…and the day before yesterday an old man died…//…I’ve seen him a lot, he had been here for years, and his wife always used to come and see him every day…*it took me*…but I’ve felt…felt sad and mournful when I see others who are suffering.

(7, man, residential care)

#### In summary

Waiting and not being respected as adults, or being treated like tasks to be performed, bring experiences of diminished dignity and are closely linked to questions about human existence and existential loneliness. The narrations about existential loneliness show similarity with Eriksson’s theory about suffering of care as the theory describes suffering when experiencing the care as non-caring and unloving due to violated dignity, condemnation, and assertion of power. Existential loneliness was not found in all three care contexts, but only in residential care and home care. Apart from waiting and being treated like a task, participants in residential care narrated how being forced to endure the suffering of others, without anywhere to escape, wakes their own suffering and triggers experiences of existential loneliness.

### Existential loneliness in relation to suffering of life

The category covers existential loneliness in relation to suffering of life and contains the participants’ narrations about their loss of close relationships, looking back on life and feeling regret, feeling guilt, a lack of meaning and purpose in life, and not feeling at home. Most of these experiences were found in all three care contexts. However, narrations about not feeling at home were only present in residential care and home care, while guilt was only found in narrations from specialized palliative care.

#### Abandoned and forgotten in the loss of close relationships

Narrations regarding existential loneliness related to suffering of life frequently concerned relationships with other people who had been important to them through life, often in connection with lost and/or broken relationships with family or close friends. The lost and broken relationships made them feel abandoned and forgotten and gave rise to existential loneliness. Such narrations were found in all three care contexts. In some cases, friends and family members had died, while in others, no one kept in touch anymore. The participants referred to how their family and friends did not keep in contact with them anymore and how this made them feel forgotten and gave rise to existential loneliness. The narrations of participants receiving *residential care* illustrate how fewer and fewer people contacted them, how no one was interested in who they were or had been, and that such experiences brought existential loneliness, while narrations from participants receiving *home care* illustrate experiences of not being interesting anymore. Such experiences arose when people who visited or called them did not seem to be interested in *them* but came because they felt obliged to come. When being asked to talk about situations that triggered existential loneliness, a woman receiving home care narrated about how a relative who called her did not call because she genuinely *wanted* to, but rather because she had a bad conscious and felt obliged:

…but today she called me, and she was in such a state of confusion, so it was ridiculous, ‘I’m so tired, I’m so annoyed and I’m so…oh, sorry I haven’t called, and it was you who called a while ago’, etc. Yes, yes, yes, said I, take it easy… but you have no use for people like that when you’re ninety years old… and standing on the last step, absolutely not.

A: What do you have in mind when you say that you have no use for people like that, what are you thinking when you are…?

… Well, then they should…if they care a bit, they should show it at the moment, that little moment they talk to me on the phone or they come here…they look at the clock and ‘now I have to drive’, it’s like…then I have it calmer when they’re not around.

(1, woman, home care)

#### Remorseful over what cannot be undone in life

Existential loneliness in relation to suffering of life emerged when the participants looked back on life and expressed remorse; this was present in narrations from all three care contexts. Their narrations covered longing for what had been, and the choices they had made throughout life. The participants referred to situations where they regretted how they had acted or *not* acted, what they had done or *not* done. Thoughts that gave rise to existential loneliness in remorse over what cannot be undone in life. There were descriptions of how they now, with their experiences of ageing and illness, would have acted differently in earlier life and how this makes them feel remorseful. A participant from *specialized palliative care* expressed how he had “thought a lot about it *afterwards*” and that he would have prioritized differently today.

…I’ve thought a lot about it afterwards, that my mother was probably very lonely. I understood it in the following way, that…when I had a bad conscience, I paid for a trip so she could travel, or I gave her…she never…needed any money, it turned out later that when she died, she had money, but that’s another matter…. well, not much, but I could give her money, but she didn’t want my money, she wanted me to come to the kitchen table and sit and talk to her for half an hour and I didn’t understand that…she wanted my company…and I solved it…it was wasted time, I thought, then it’s better to give her something and there I was wrong…

(19, man, specialized palliative care)

#### Lack of meaning and purpose in life

Narrations covered how life was becoming increasingly uninteresting, spirit-poor, and meaningless and how such feelings led to existential loneliness. Such experiences were present in all three care contexts. Lack of meaning and purpose emerged when the participants did not feel valuable. Participants who received *residential care* described moments when they felt that they were not important to anyone anymore. A woman expressed this by saying “they would probably be happy to get rid of me” and described how she had to “be seated” and felt like a prisoner in the residential care facility where she lived:

…then I have to sit here like a prisoner. A: You feel like a prisoner? Yes, I can say that I do, in the middle of everything…They would probably be happy to get rid of me…Yes, they have so much to do anyway. A: Don’t you feel that you’re important here? No, they just have trouble with me.

(14, woman, residential care)

In addition, lack of meaning and purpose in life was expressed when participants who received residential care referred to living in “the waiting room for death”. Residential care was seen as their last station in life from which no one comes out alive. A woman told how everyday life no longer had any purpose and meaning but rather was death’s waiting room. Such experiences involved feelings of existential loneliness:

…Nobody gets well here, nobody leaves…what can I say…nobody gets out of here alive…It’s the waiting room for death…

(12, woman, residential care)

#### Not feeling at home

Experiences of not feeling at home and a lack of belonging were only present in narrations from residential care and home care. These were caused by having to move, often due to a need for care. Participants who received *residential care* had to live together with others, but as they knew few of them, they felt alone and excluded even though there were people around. A man receiving residential care expressed a lack of belonging in his description of how he lives together with 10 other people, but cannot talk to any of them:

… most of them can’t talk at all, so we have no exchange among ourselves…//…there are eleven of us here and I can’t have a conversation with anyone…

(7, man, residential care)

Even though the participants understood, had accepted, and sometimes had wanted to move to another home, existential loneliness was experienced when not feeling at home at the place where they now lived. Some of those who received *home care* and had recently moved to an apartment experienced existential loneliness, like this man who did not feel at home and wished that he had never had to move:

That’s where I felt at home and that’s where I thought we were going to live for the rest of our lives…that’s what it was…a very nice place…so it…is incredible…it was a wooded hill, one hectare, it was a thousand square metres…and we felt at home there, really…//…but this time we had to move…It’s the only time

(21, man, home care)

#### Guilt for how they have lived their lives

Existential loneliness in relation to suffering of life was also expressed in terms of guilt and was only present in narrations from specialized palliative care. Guilt was expressed in narrations about how the participants had lived their life; for example, feeling guilt over smoking, a choice earlier in life which had now made them sick. The narrations about guilt were, however, often intertwined with narrations about how the guilt was dispelled in a care context where such feelings were allowed. A man in specialized palliative care tells how he previously felt guilty about how he had lived his life, but now his guilt had drained away:

… and they [the professionals] asked me if I still smoked. Yes, but very, very little, but basically a stop… Then he says like this… [the physician] ‘Now it’s like this in plain language. You’re in such bad shape, can you … have your cup of coffee and your cigarette and care for yourself, do it’…well, then that feeling disappeared…//… and then to…try to sneak away… hypocrisy…//…to let go of that damned need for concealment…that it brings with it.

(17, man, specialized palliative care)

Guilt was also described in relation to injustice in the world, and participants described how they had seen people starving in other parts of the world while they themselves had lived in abundance. Such thoughts came to mind for some of the participants at this time in their lives and made them feel guilt.

#### In summary

Situations where people experience the lack of former close relationships, that their life has no meaning and that they themselves do not have any meaning for anyone else anymore make people feel lonely in an existential sense. The narrations about existential loneliness show similarity with Eriksson’s theory about suffering of life in relation to what it means to live and be a human being among other human beings. No matter the care context, existential loneliness in relation to suffering of life was found in older persons’ narrations. Existential loneliness connected to guilt and remorse over choices one has made or *not* made throughout life were, however, only found in specialized palliative care, while narrations about not feeling at home were only present in residential care and home care. Such experiences raised questions about human beings’ existence.

## Discussion

The result reveals circumstances and situations where existential loneliness arises in relation to suffering, i.e., suffering of illness, suffering of care, and suffering of life in different care contexts. Based on the older persons’ narrations, existential loneliness was solely described in negative terms. The three dimensions of suffering are based on Eriksson’s (1994/2006) theoretical framework about *the suffering human being* and were used as an analytic grid in the present study. We found similarities between our findings based on interviews with older people about existential loneliness, i.e., a deep feeling of loneliness, and Eriksson’s (1994/2006) theory about suffering. In her theory, Eriksson addresses loneliness as a central aspect of suffering; she describes, for example, that being excluded from community may entail suffering, which also our analysis revealed. Here, we see similarity with what we refer to as existential loneliness. According to Eriksson, loneliness arises when people are deprived of something that is or has been important in their lives, such as the loss of a partner or friends and family who no longer come to visit based on their free will but rather on a sense of duty. Being deprived of something that is important to a person is seen by Eriksson as a circumstance that causes loneliness and the deepest suffering is caused by deprivation of dignity. There are also other similarities with Eriksson’s theory of suffering, findings about situations and circumstances that can trigger existential loneliness: violated dignity, guilt, condemnation, unloving, not feeling welcome, or not being taken seriously. From our study, it also emerged that central to the experience of existential loneliness was a physical body that no longer functioned, a body that betrayed and was experienced as a loss. Here too we noticed links to Eriksson’s theory, what she refers to as suffering of illness, i.e., suffering in relation to illness or treatment. Accordingly, to the body of knowledge, this study adds that suffering and existential loneliness is interrelated for frail older people in need of long-term care and that existential loneliness might arise because of situations and circumstances in the present but also when memories from previous life come to mind. It also seems that some of the situations and circumstances that trigger existential loneliness are the same regardless of care context while others are more tied to a specific care context. Yalom (1980) uses a valley as a metaphor when describing the inevitable stages of life that we must go through as humans. Being in the valley involves experiences such as existential loneliness. This study shows that suffering of illness, suffering of care, and suffering of life are examples of such valleys mentioned by Yalom. The interpreted narrations were from persons receiving home care, residential care, or specialized palliative care. The division into these three contexts was based on where the participants mainly lived their daily lives and received long-term care. Regardless of the care context, existential loneliness was found in narrations about suffering of life and suffering of illness while existential loneliness in suffering of care was not found in the specialized palliative care context.

Care situations where the dignity of a person is threatened or where power is exercised create a risk of existential loneliness. Participants receiving residential care and home care described care situations where they felt reduced to a task which needed to be performed, felt they were not encountered like an equal adult, and felt like part of a piece of machinery, leading to existential loneliness in relation to suffering of care. Health care professionals are performing their role in organizations where often a reductionistic and illness-centred perspective remains in the foreground and the wholeness of a person tends to be in the background (Eriksson, 1994/2006). Such a care system poses the risk that the caregivers are deprived of their opportunity to see the whole situation and thus, unintentionally, cause existential loneliness in those they care for. There are a number of studies that show how professionals in health care are prevented from performing work to meet the needs of the people they care for, and that this in turn negatively affects their own well-being (Collet et al., 2018; Schön Persson et al., 2018). However, diminished dignity and assertion of power were not prominent in the narratives from participants receiving specialized palliative care. The palliative care philosophy is largely in line with the person-centred approach that is receiving increasing attention in the contemporary view of what characterizes good care. A core value in palliative care and person-centred care is to care for the totality of the person, namely *for* the person, *by* the person and *with* the person. A necessity is to strive for a trustful relationship and to balance the power between the receiver of care and the caregiver (McCormack & McCance, 2017; Radbruch & Payne, 2010). Eriksson (1994/2006) highlights the importance of confirmation and that alleviating a person’s suffering means not violating dignity, not condemning or abusing power. According to a previous study, a trustful relationship between the professionals and the persons in need of care was found to be a prerequisite for the professionals to encounter existential needs (Sundström et al., 2018). The existentialist philosopher Paul Tillich states that humans need to be seen and confirmed in the eyes of another person, otherwise people will be transformed into things, into pieces (Tillich, 1952/2014). Such a relationship is built when encountering each other as equals (McCormack & McCance, 2017) and as the philosopher Martin Buber states, as an encounter between *I* and *thou* and, not *I* and *it* (Buber, 1923/2013). McCormack and McCance (2017) describe conditions for the relationship as *be in time* and *be present*, i.e., to be there for another person when that person needs it. A person-centred approach also promotes *being in relation*, which covers the efforts to connect with oneself, other persons, and contexts and which is expressed in the way one provides care (doing), talks about it (knowing), and is (being) (McCormack & McCance, 2017). In addition, for health care professionals to have the possibility to be in time, be present and strive for being in relation together as an equal with the persons they provide care for, they need organizational support—such as leaders who encourage a person-centred approach that preserves the persons’ dignity and equality of power (McCormack & McCance, 2017; Söderman et al., 2021).

Existential loneliness in relation to suffering of life was found in narratives from persons receiving residential care and home care when not feeling at home in the place they live. The loss of one’s former home could cause experiences of loneliness. According to Eriksson (1994/2006) loneliness arises when persons are deprived of something of great importance to them. Such loneliness can become unbearable. An expectation in general among people is, however, that if you move to a residential home, you will not have to be alone. Although this in itself it is true—you are not alone at a residence as there are always people around—you can feel just as lonely, or maybe even lonelier after the move if you feel odd, different or have to wait for care. Another reason for experiencing existential loneliness in residential care is if you have to put up with being in a place where others around are suffering, badly treated, or uncared for. The ability to suffer with other people is deeply human-rooted and is about our human ability to feel compassion (Eriksson, 1994/2006). One reason why persons receiving residential care were more likely to experience existential loneliness when people around them were uncared for can be related to ageing, as they were considerably older than those being cared for in palliative care. Tornstam’s theory of gerotranscendence explains that human beings develop their deeper feelings throughout life, and notions of life and existence, such as having to relate to other people’s suffering, become more important, especially in the latest phase of life (Tornstam, 1978/2018). Throughout life, there is also a deep longing for relatedness to places, people, and material belongings (Carr & Fang, 2021; Lyberg et al., 2013). Other studies have shown the need for relatedness. Österlind et al. (2017) pointed out how older people’s lives were characterized by feelings of aloneness in an unfamiliar place, which contributed to a sense of existential loneliness. Their study highlights the importance of supporting people in their transition before, during and after relocating to a nursing home (Österlind et al., 2017). As existential loneliness was found not only in narratives from persons who had moved to residential care but also among those who received home care and did not feel anchored and at home in the place they lived, the present study shows that support is needed when moving to any *new place*. A person-centred approach involves *being in a social context* which involves identifying and linking things that are important in the context of the world the person lives in (McCormack & McCance, 2017); this may in turn lead to alleviating suffering of life. A person-centred culture needs to consider the place where people live and are cared for and how the provided care enables, or prevents, feelings of belonging and relatedness. A person-centred culture means promoting opportunities for people to feel safe and at home in the context in which they live (McCormack & McCance, 2017).

Regardless of the care context in which the care is provided, there is a risk of experiencing existential loneliness in relation to suffering of life when feelings of guilt and shame arise. However, our findings show that the origins of guilt and shame can vary. While feelings of shame about who one has become were represented in all three care contexts, feelings of guilt over having, for example, caused the disease themselves were only expressed in palliative care. Although we did not directly ask participants about their medical conditions, we can assume that cancer was the most common diagnosis for those receiving specialized palliative care (Sjöberg et al., 2021). The findings show that shame was experienced in relation to suffering of illness caused by illness and treatment, while guilt was experienced in relation to suffering of life connected to how they had lived their life, and the choices made or *not* made earlier in life. According to Sartre (1947/2007) guilt arises when one feels bad about an action or something one has done, while shame is about the person one is—i.e., the self and identity. One way to help people overcome shame and guilt is by creating an open, trustful, allowing atmosphere where condemnation is avoided. To ease suffering of illness, Eriksson (1994/2006) describes the importance of awareness of the ethics of caring, and good care and a person-centred approach involves *being with self*, i.e., being aware of personal values to understand the values of others (McCormack & McCance, 2017). An action of caring is to ease another person’s feelings of guilt and shame (Eriksson, 1994/2006) and person-centredness fosters an allowing and open atmosphere for the other person’s different needs (McCormack & McCance, 2017).

## Methodological considerations

A limitation of the present study is that there is no clear consensus on the concept of existential loneliness. In addition, there are intertwinings with other forms of loneliness such as social and emotional loneliness. For example, the category *Abandoned and forgotten in the loss of close relationships* might overlap with social loneliness. However, as described in the introduction, social loneliness is understood as a longing for companionship. In focusing on existential loneliness, we strove, however, to understand participants’ experiences of feeling abandoned and forgotten, existential experiences linked to vulnerability and human existence. Such experiences were interpreted as existential loneliness. The difficulty of distinguishing existential loneliness from social and emotional loneliness may nevertheless be seen as a weakness and a challenge to the *credibility* of our data (Guba, 1981). To counter this possibility, we introduced the concept to the participants in the written information and to increase the probability that the participants narrated about existential loneliness we started the interview by talking about loneliness in general and then deepened the interview to ask about existential loneliness in particular. In accordance with Yalom (1980), we assumed that all the participants had experiences of existential loneliness. As we strove to be open minded and wanted a broad range of narrations to cover existential loneliness, every participant got the question about existential loneliness even if they had stated that they did not experience loneliness *at present*. The participants were encouraged to talk about their own experiences. However, some participants talked about other people who they perceived as lonely in an existential sense. Those narrations were captured in the category *Exposed and forced to seeing and hearing others suffering*, that is, when they perceived other people suffer, this in turn gave rise to their own existential loneliness. In addition, during the analysis process, we strove to only include narrations that were judged to relate to the existential dimension of loneliness. The interviews were performed by two persons, one junior researcher and one senior researcher, who were both familiar with conversations about existential concerns. To safeguard *confirmability* (Guba, 1981), we continuously discussed our preunderstanding. Two of us have a background as registered nurses in the field of geriatric care and palliative care and one of us has a background as a researcher in the field of geriatric care. One of us (IB) was involved in the interviews and therefore had pre-knowledge regarding the specific interview situations, while the other two of us were not affected by this. We all took part in the analysis and discussed the categories until an agreement was reached. Regarding *dependability* (Guba, 1981) our analysis process is described in the method, in the text and in tables, and extracts from raw data have been presented in the findings. However, as this paper represents a secondary analysis of previously collected data, the selection procedure is described more in detail in a previous article (Sjöberg et al., 2018). Concerning the *transferability* (Guba, 1981) of the findings, the participants in this study came from a Swedish health care context. To illustrate for readers who are unfamiliar with the Swedish care context and thus make it possible for readers to transfer the results, the three different care settings are described in the method section. The participants were identified within the different contexts with a variation in age, gender, and care context which might strengthen the transferability to several care settings.

## Conclusion

Existential loneliness was mirrored in relation to Eriksson’s theory about the suffering human being and our findings show similarities with the theory. To the body of knowledge, this study adds that suffering and existential loneliness are interrelated for frail older people in need of long-term care. The results show that existential loneliness in relation to suffering of care was prominent in residential care, while it was not found in specialized palliative care. These two forms of care context have different resources. In specialized palliative care, there is access to different professionals such as registered nurses and physicians working in teams, professionals with a clear assignment to relieve suffering. In residential care, most of the staff are nurse assistants with varying levels of education and with the main assignment to provide support in everyday life. One conclusion is that level of education, knowledge, and the possibility of cooperation between different professional groups have an impact on the ability to encounter the care needs of older people and thus on the possibility to reduce suffering of care and ease existential loneliness. Regardless of care context, there are situations and circumstances when people experience suffering that all professionals need to pay attention to as these can trigger existential loneliness: feeling dependent, lack of close relationships, experiences that life has lost its meaning, not feeling important for anyone else, as well as feeling guilt for whom one has become. Other aspects are more linked to specific care contexts. In residential care and home care, professionals need to pay special attention to unnecessary waiting in uncertainty, not feeling at home, and not being encountered like an adult, as this can trigger existential loneliness. Professionals in specialized palliative care need to be particularly sensitive to people who feel guilt and remorse over how they have lived their earlier lives. Finally, in institutional care, which in this study is represented by residential care, it is inevitable that residents will witness other older people expressing suffering. Such situations can trigger existential loneliness among those who witness the situations. In order to avoid unnecessary suffering, it is important to have knowledge and insight into circumstances and situations that make older people experience existential loneliness. Our endeavour with this study has been that the findings should be used to develop a person-centred care, i.e., a care where all the needs of the older persons, including the existential ones, are met. Since different care contexts have different conditions, there is no single way to reach this. We therefore propose that the findings will be used as a basis for reflective discussions in working groups on how person-centred care in one’s own workplace could be developed. In order to conduct such discussions, it is important that the necessary conditions are provided by society as a whole as well as within the organization.
